# Short-term and long-term efficacy of accelerated transcranial magnetic stimulation for depression: a systematic review and meta-analysis

**DOI:** 10.1186/s12888-024-05545-1

**Published:** 2024-02-07

**Authors:** Ruifeng Shi, Zuxing Wang, Dong Yang, Yujie Hu, Zhongyang Zhang, Daotao Lan, Yihan Su, Yunqiong Wang

**Affiliations:** Sichuan Provincial Center for Mental Health, Sichuan Provincial People’s Hospital, School of Medicine, University of Electronic Science and Technology of China, Chengdu, China, No. 32, West 2nd Section, 1st Ring Road, 610031 Chengdu, Qingyang District China

**Keywords:** Accelerated transcranial magnetic stimulation, Major depressive disorder, Short-term efficacy, Long-term efficacy

## Abstract

**Background:**

In recent years, accelerated transcranial magnetic stimulation (aTMS) has been developed, which has a shortened treatment period. The aim of this study was to evaluate the efficacy and long-term maintenance effects of aTMS in patients with major depressive disorder (MDD).

**Methods:**

We systematically searched online databases for aTMS studies in patients with MDD published before February 2023 and performed a meta-analysis on the extracted data.

**Results:**

Four randomized controlled trials (RCTs) and 10 before-and-after controlled studies were included. The findings showed that depression scores significantly decreased following the intervention (SMD = 1.80, 95% CI (1.31, 2.30), *p* < 0.00001). There was no significant difference in antidepressant effectiveness between aTMS and standard TMS (SMD = -0.67, 95% CI (-1.62, 0.27), *p* = 0.16). Depression scores at follow-up were lower than those directly after the intervention based on the depression rating scale (SMD = 0.22, 95% CI (0.06, 0.37), *p* = 0.006), suggesting a potential long-term maintenance effect of aTMS. Subgroup meta-analysis results indicated that different modes of aTMS may have diverse long-term effects. At the end of treatment with the accelerated repetitive transcranial magnetic stimulation (arTMS) mode, depressive symptoms may continue to improve (SMD = 0.29, 95% CI (0.10, 0.49), *I*^*2*^ = 22%, *p* = 0.003), while the accelerated intermittent theta burst stimulation (aiTBS) mode only maintains posttreatment effects (SMD = 0.01, 95% CI (-0.45, 0.47), *I*^*2*^ = 66%, *p* = 0.98).

**Conclusions:**

Compared with standard TMS, aTMS can rapidly improve depressive symptoms, but there is no significant difference in efficacy. aTMS may also have long-term maintenance effects, but longer follow-up periods are needed to assess this possibility.

**Trial registration:**

This article is original and not under simultaneous consideration for publication. The study was registered on PROSPERO (https://www.crd.york.ac.uk/prospero/) (number: CRD42023406590).

**Supplementary Information:**

The online version contains supplementary material available at 10.1186/s12888-024-05545-1.

## Introduction

According to Liu et al. (2020), there were 162 million major depressive disorder cases in the world in 1990 and 241 million cases in 2017 [1]. China contains 18.4% of the world’s population and 21.3% of major depressive disorder (MDD) cases, indicating that depression imposes a substantial burden of disease [2]. According to the Global Burden of Disease Study, major depression is a leading cause of disability, ranking second in terms of its contribution to the disease burden [3].

Despite the availability of various treatment options for MDD, such as medication and psychotherapy, patients remain susceptible to relapse [4]. Transcranial magnetic stimulation (TMS), a noninvasive and safe method of brain stimulation, is among the numerous alternative therapies available for treating MDD. TMS, either high-frequency or low-frequency, has a slower and lower effect on synaptic plasticity than theta burst stimulation (TBS), a recently developed type of TMS that imitates the endogenous hippocampal theta pattern [5, 6]. The two main components of TBS are continuous theta burst stimulation (cTBS), which has inhibitory effects, and intermittent theta burst stimulation (iTBS), which has excitatory effects. Repetitive transcranial magnetic stimulation (rTMS) therapy involves 10 Hz stimulation of the left dorsolateral prefrontal cortex once per day for 4–6 weeks to improve depressive symptoms [7]. In addition, recent meta-analyses have shown that TMS, particularly bilateral TBS, can reduce depressive symptoms in the treatment of MDD [8]. Unfortunately, extended treatment periods (10–20 sessions for improvement in depressive symptoms, followed by 6 weeks of treatment or longer to enhance response rates) with TMS are necessary [9]. Therefore, such treatment is not recommended for patients with severe symptoms requiring rapid treatment.

In recent years, researchers have proposed multiple daily TMS sessions to rapidly improve depressive symptoms. Accelerated transcranial magnetic stimulation (ATMS) includes accelerated repetitive transcranial magnetic stimulation (arTMS) and accelerated intermittent theta burst stimulation (aiTBS). Holtzheimer et al. showed a significant effect of aiTMS on day 3 of treatment, with a 47% decrease in the mean Hamilton Depression Rating Scale (HDRS) score after administering 15 TMS sessions over 2 days; the effect persisted at the 6-week follow-up [10]. Cole et al. carried out two aTMS sessions in 2020 and 2021 to treat patients with major depressive disorder; 10 daily sessions of iTBS were administered for 5 consecutive days, and the results showed that the response rate reached approximately 90% [11, 12]. However, at the fourth week of follow-up, the depression scores rebounded. Somnez et al. conducted an inaugural meta-analysis of aTMS for depression treatment. The authors posited that the aTMS protocol may offer a more efficient and accessible treatment alternative for depression than standard TMS protocols [13].According to Chen et al.‘s meta-analysis, accelerated high-frequency (HF) rTMS had an effect equivalent to that of standard rTMS, although only four studies were included [14]. Cai et al.‘s meta-analysis involving five studies revealed that aiTBS is more effective at treating major depressive episodes in patients with MDD or bipolar disorder [15]. In a smaller-scale study by Williams et al., accelerated high-dose iTBS was employed for depression treatment. Despite most patients experiencing remission, all participants relapsed within two weeks posttreatment [16]. Notably, no studies have explored the long-term efficacy of this treatment. Consequently, the long-term effectiveness of aTMS remains uncertain. It has been demonstrated that gender-specific variations in brain regions exist in patients with depression [17], and the efficacy of TMS is influenced by age and baseline depression severity [18–20]. Thus, we included age, sex, and baseline severity as variables in our meta-regression analysis.

## Methods

The effectiveness of aTMS in treating depression over the short and long term was thoroughly reviewed. This review was submitted to PROSPERO and adhered to the PRISMA guidelines [21]. (ID number: CRD42023406590).

### Search strategy and selection criteria

The literature was retrieved from the PubMed, Web of Science, and Embase databases from inception through February 2023. The search terms for depression included “depression”, “depressive disorder”, and “treatment-resistant depression”. The aTMS search terms included “accelerated repetitive transcranial magnetic stimulation”, “accelerated transcranial magnetic stimulation”, “accelerated rTMS”, “accelerated TMS”, “accelerated iTBS”, “accelerated cTBS”, and “Stanford Neuromodulation Therapy”. The complete search terms used are listed in the Supplementary Material. Another author conducted a peer review of the search strategies. The inclusion criteria were as follows: (1) studies including patients diagnosed with major depressive disorder according to the DSM-IV, DSM-V, or ICD-10 criteria; (2) studies in which aTMS was used in the experimental group; (3) studies with a randomized controlled trial (RCT) or before-and-after controlled trial design (studies with a crossover design were evaluated before crossover only); (4) studies in which a scale was used to evaluate depressive symptoms before and after the intervention; and (5) studies published in English. The exclusion criteria for articles were as follows: (1) case reports of a single case and (2) duplicates.

### Quality assessment

To evaluate the potential for bias, the Cochrane Collaboration’s risk of bias tool was used by individual reviewers involved in the data extraction. The tool includes the following domains: random sequence generation, allocation concealment, blinding of result assessment (for detection bias), inadequate outcome data (for attrition bias), selective outcome reporting (for reporting bias), and other biases. The danger of bias was lowest in the first two domains and highest in the last two domains. The answer options for each domain were “definitely yes”, “probably yes”, “definitely no”, and “probably no”. To evaluate the validity of each study, sensitivity analysis was also carried out using the “leave-one-out” method. Publication bias was estimated using the Egger test with Stata 15.1 software, and if publication bias was present in the results, we estimated the effect of publication bias on the results by the “trim and fill method” method.

### Data extraction

The titles and abstracts of the retrieved articles were filtered by the writers using NoteExpress and EndNote X9. After carefully reading the eligible studies that satisfied the inclusion requirements, two reviewers worked independently to extract information from the studies. A third reviewer was consulted in the event of a dispute. Data on the name of the initial author, nation of origin, year of publication, baseline sample size, average participant age and sex, disease type, number of daily aTMS sessions, TMS methodology, and scale choice were collected. The mean depression scores at baseline, immediately following the intervention, and at follow-up, as well as the follow-up length and the number of participants who completed the analysis, were included as outcome measures. For crossover control experiments, only precrossover data were included.

### Data analysis

The meta-analysis was conducted using Review Manager 5.4 and STATA 15.1. Individual study intervention effects were combined, and the treatment effect size for each study was calculated based on the standardized mean difference (SMD) between pre- and posttreatment depression scores. These effect sizes were then combined to arrive at an overall treatment effect size for the described comparison. Depression scales were prioritized over the most commonly used scales (e.g., the HDRS and the Montgomery-Asberg Depression Rating Scale (MADRS)). If a self-administered scale was used in the study, the scale that was used to draw the main conclusions of the article was selected. To check for heterogeneity in all meta-analyses, the Cochrane Q *p* value and *I²* statistic were used. Bonferroni correction was used for multiple comparisons, and the critical *p* value was 0.05/8 for meta-analysis. If *I*^*2*^ was ≥ 50%, significant heterogeneity was indicated, and a random effects model was used. Otherwise, a fixed-effects model was used. For the main results of the article we have performed meta-regression analysis. Two subgroup analyses were conducted using the available data to evaluate the efficacy and long-term effects of different aTMS modalities, namely, the arTMS modality and the aiTBS modality, for treating depression.

## Results

### Study characteristics

**Fig. 1:**
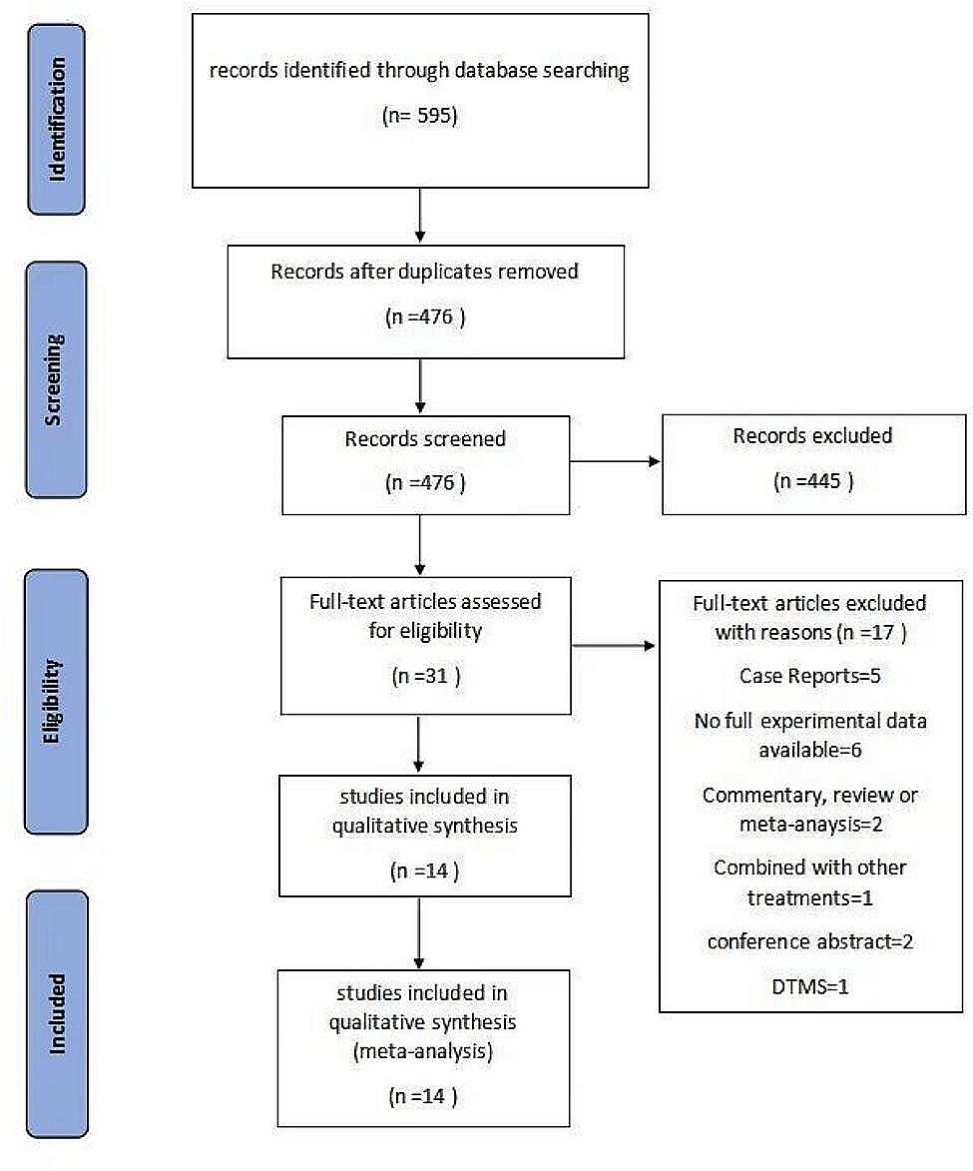
Diagram of the preferred reporting items for systematic review and meta-analysis (PRISMA)

A total of 595 studies were initially retrieved from the databases. After removing duplicates, we proceeded to screen the titles, abstracts, and full texts to determine eligibility (Fig. 1). Ultimately, 14 studies met the inclusion criteria—4 RCTs (3 comparing aTMS to standard TMS and 1 comparing aTMS to pseudostimulation) and 10 before-and-after control studies. For more information, refer to Table 1. We assessed the quality of the articles to evaluate the risk of bias (refer to Supplementary Fig S3). All of the stimulation targets in the studies included were dorsolateral prefrontal cortex (DLPFC), Four studies utilized personalized precision localization [11, 22–24], five studies employed the electroencephalogram (EEG) F3/F4 localization method [25–29], and five studies utilized the 5–6 cm anterior localization of the primary motor cortex [10, 30–33]. Four studies had a one-month follow-up duration [11, 22, 26, 27], and the follow-up periods varied from three weeks [33] to three months [31]. A six-week follow-up period was used in one study [10]. A two-month follow-up period was used in two of the studies [29, 32], however, the follow-up duration was not included in the other study [24]. The most commonly used depression severity rating scales in these studies were the HDRS, the MADRS, the Quick Inventory of Depressive Symptoms (QIDS-C), and the Korean Quick Inventory of Depressive Symptoms Self-Report (KQIDS-SR). Four of the studies were conducted in the USA, 3 were conducted in Australia, 2 were conducted in Belgium, 1 was conducted in China, 1 was conducted in Singapore, 1 was conducted in Canada, 1 was conducted in Italy, and 1 was conducted in Korea.

**Table 1:** Characteristics of the 14 included studies

Author (year)	Country	Study Design	Patient Sample Accelerated TMS(N/Age/ M/ F)	Standard TMS or Sham TMS(N/Age/ M/ F)	TMS schedule	Stimulation site		rTMS Parameters				Main diagnosis	Treatment outcome measure used
							Frequency	MT% TMS model	Single pulse/Total pulses	session Train/lengthInte r-train interval	Inter-session interval		
Zhang: et al.:2022	China	Self-controlled study	31/(31.5± 3.4)/0/31	31/(31.7± 6.3)/0/31	10sessions/day x 5days	L- DLPFC	n/a	90% iTBS	1800/90,000	Not mentioned	50 min	Postpartum depression (PPD)	HAM- D EPDS
Baeken: et al.:2020	Belgium	RCT Cross-controlled trials	22/(40.09 ± 11.45)/6/ 16	24/(42.75± 12.24)/7/ 17	5sessions/day x 4days	L- DLPFC	n/a	110% iTBS	1620/32,400	2s/8s	15 min	TRD	HAM- D
Tan: et al.:2021	Singapore	Self-controlled study	60/	n/a	4sessions/day x 5days	L- DLPFC	5HZ	120% rTMS	3000/60,000	8s/ 12s	/	MDD	MADRS
Cole: et al.:2020	USA	Self-controlled study	21/(44.86 ± 17.21)/9/ 12	n/a	10sessions/day x 5days	L- DLPFC	n/a	90% iTBS	1800/90,000	2s/8s	50 min	19MDD 2BD- II	MADRS HAM- D
Frey: et al.:2020	USA	Self-controlled study	6/(66.33± 4.97)/5/ 1	n/a	5sessions/day x 4days	L- DLPFC	20HZ	110% rTMS	1560/31,200	2s/ 12s	10- 15 min	Post-stroke depression (PSD)	HAM- D
Miron: et al.:2021	Canada	Self-controlled study	48/(41.8± 12.2)/21/27	n/a	Week 1: 6sessions/day x 5days Week2: 20-25sessions	R- DLPFC	1HZ	120% rTMS	360/ 18,000- 1 9800	60s/30s	50 min	MDD	BDI- II HRSD- 17
Cantù: et al.:2021	Italy	Self-controlled study	4/(53.5± 11.09)/2/2	n/a	Week 1: 3sessions/ day x 3days Week 2: 3sessions/day x 2days Week 3: 3sessions/day x 1days	L- DLPFC	n/a	80% iTBS	600/ 10,800	2s/ 12.3s	15 min	TRD	HAM- D HAM-A MADRS
Kim: et al.:2021	Korea	RCT	21/(45.1± 13.5)/4/ 17	22/(43.9± 10.9)/3/ 19	Accelerated rTMS:5sessions/day x 3days Conventional rTMS: 1sessions/day x 15days Accelerated group:	L- DLPFC	10HZ	110% rTMS	3000/45,000	5s/25s	30 min	MDD	KQIDS-SR KQIDS-C
Fitzgerald: et al.:2018	Australia	single blind RCT	58/(48.2± 14.4)/25/33	57/(49.9± 13.3)/24/33	Week 1: 3sessions/ day x 3days Week 2: 3sessions/day x 2days Week 3: 3sessions/day x 1days Standard Group: 1sessions/day x 20days(4 weeks)	L- DLPFC	10hz	120% rTMS	3500/63,000	4.2s/ 15s	15-30 min	MDD	HDRS MADRS BDI SSI
Holtzheimer 3rd: et al.:2010	USA	Self-controlled study	14/51 (21–74)/9/5	n/a	Day 1: 5sessions Day 2: 10sessions	L- DLPFC	10hz	100% rTMS	1000/ 15,000	5s/25s	50 min	TRD	HDRS24 HAM-A BDI
Tor: et al.:2016	Australia	Self-controlled study	7/36 (23–53)/4/3	n/a	Day 1: 1sessions Day 2–3: 5sessions Day 4–10: 1sessions	R- DLPFC	1HZ	120% rTMS	900/ 16,200	/	30 min	4 BD- II 3 Depression	MADRS BDI- II
Desbeaumes Jodoin: et al.:2019	USA	open-label	54/43.8 (9.0: 25 − 59)/26/28	19/71.0 (8.3: 60 − 89)/10/9	2sessions/day x 15days	L- DLPFC	20HZ	110% rTMS	3000/45,000	5s/25s	/	MDD	MADRS HAM-A
Baeken: et al.:2017	Belgium	RCT Cross-controlle d trials	44/(41.0± 12.0)/12/32	n/a	5sessions/day x 4days	L- DLPFC	n/a	110% iTBS	1620/32,400	2s/8s	15 min	MDD	HDRS SSI BHS
Chen: et al.:2021	Australia	single blind RCT	103/(49.14 ± 15.77)/33/70	84/(48.67± 16.06 )/35/49	Accelerated TBS: Week 1:11 sessions Week 2:9 sessions Standard rTMS:1sessions/day x 20days(4 weeks)	ATBS: R- DLPFC for cTBS followed by L- DLPFC for iTBS SrTMS:L- DLP FC	ATBS: 3 x 50 Hz pulses at 5 Hz bursts SrTMS:1 0HZ	120%	ATBS:1200/ 24,000 SrTMS:3000/ 60,000	ATBS:/ SrTMS:4s/26s	ATBS:15 min SrTMS:/	MDD	QIDS-C16 QIDS-SR16

Abbreivations: TMS: Transcranial magnetic stimulation; aTMS: accelerated transcranial magnetic stimulation; SrTMS: Standard repetitive transcranial magnetic stimulation; MT: motor threshold; iTBS: intermittent theta burst stimulation; cTBS: continuous theta burst stimulation; L-DLPFC: left dorsolateral prefrontal cortex; R-DLPFC: Right dorsolateral prefrontal cortex; EPDS: Edinburgh Postnatal Depression Scale; HAMD: Hamilton Rating Scale for Depression; MADRS: Montgomery Åsberg Depression Rating Scale; MDD: Major depressive disorder; TRD: treatment-resistant depression; BD: Bipolar Disorder; HAM-A: Hamilton Rating Scale for Anxiety; BDI-II: Beck Depression Inventory II; HRSD-17: 17-item Hamilton Rating Scale for Depression; KQIDS-SR: Korean Quick Inventory of Depressive Symptomatology Self-reported; KQIDS-C: Korean Quick Inventory of Depressive Symptomatology Clinician administered; SSI: scale of suicidal ideation; BDI: Beck depression inventory; BHS: Beck Hopelessness Scale; QIDS-SR16: Quick Inventory of Depressive Symptomatology-Self-Rated Version; QIDS-C16: Quick Inventory of Depressive Symptomatology-Clinician Rated Version; SSI: Scale for Suicidal Ideation; n/a: not applicable; RCTs: randomized controlled trials

### Results of the meta-analysis

#### Effects of aTMS on depressive symptoms in patients with MDD

**Fig. 2:**
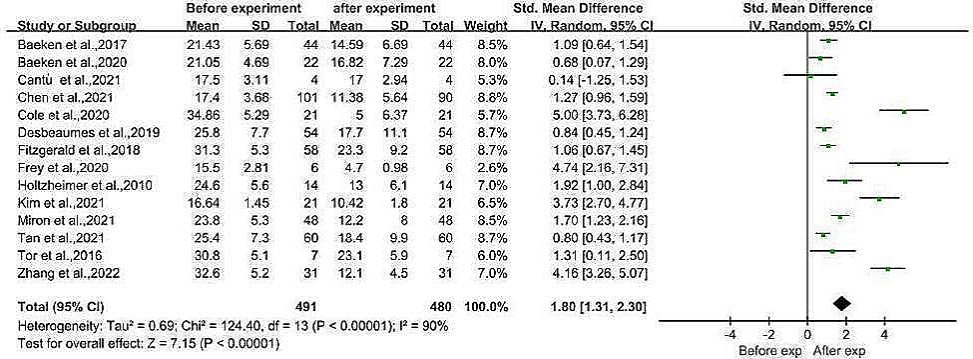
Meta-analysis of the effect of aTMS on depressive symptoms in patients with MDDAbbreviations: aTMS: accelerated transcranial magnetic stimulation, MDD: major depressive disorder

**Fig. 3:**
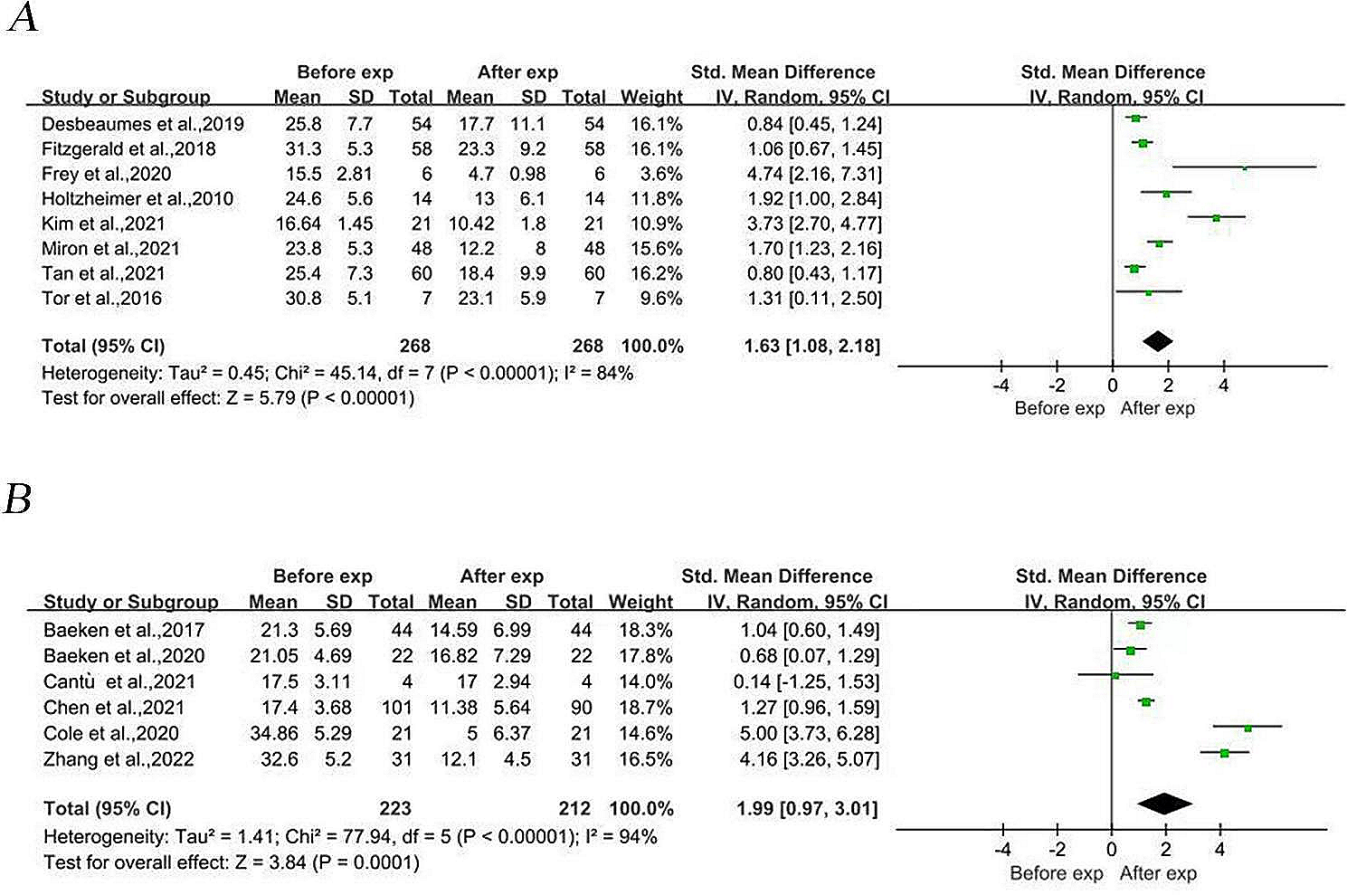
Subgroup meta-analysis comparing the effects of different aTMS models on depression severity in the pre- and post-experimental period. 3 A: Subgroup meta-analysis of the rTMS model group. 3B: Subgroup meta-analysis of the iTBS model groupAbbreviations: rTMS: Repetitive transcranial magnetic stimulation, iTBS: intermittent theta burst stimulation, aTMS: accelerated transcranial magnetic stimulation

Four RCTs and 10 before-and-after controlled trials (including 491 patients with major depression before the intervention and 480 patients with MDD after the intervention) were combined in a meta-analysis. When preintervention and postintervention participants were compared with controls, the findings demonstrated that the postintervention depression rating scale score was significantly lower than the preintervention depression rating scale score (SMD = 1.80, 95% CI (1.31, 2.30), *I*^*2*^ = 90%, *p* < 0.001; Fig. 2). Egger’s test revealed publication bias (Egger’s test, *t* = 2.76, *p* = 0.017). Meta-regression analyses were performed to assess the effects of age, sex and baseline depression severity on treatment outcomes. The findings indicated that while the male-to-female sex ratio had a significant positive effect on the outcome, i.e., the greater the male-to-female ratio was, the greater the improvement (*p* = 0.003, *z* = 2.96), age and baseline depression severity did not significantly affect the treatment outcome (effect of age: *p* = 0.587, effect of baseline severity: *p* = 0.739). We performed a subgroup meta-analysis, dividing the 14 included studies into two groups based on the modality of TMS used, the arTMS modality and the aiTBS modality. Subgroup meta-analysis revealed significant differences in both the arTMS (SMD = 1.63, 95% (SMD = 1.63, 95% CI (1.08, 2.18), *I*^*2*^ = 84%, *p* < 0.001; Fig. 3A) and the aiTBS modality subgroups (SMD = 1.99, 95% CI (0.97, 3.01), *I*^*2*^ = 94%, *p* < 0.001; Fig. 3B).

#### Comparison of depression severity before the experiment and at the follow-up

**Fig. 4:**
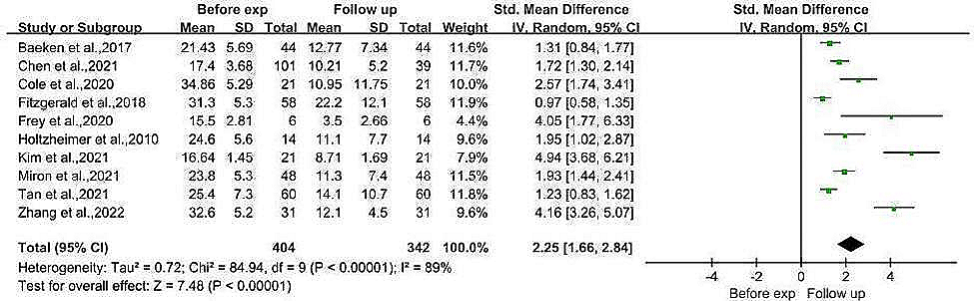
Meta-analysis of the severity of depression before the experiment and at the follow-up

Three RCTs and seven before-and-after controlled trials with a pretest sample size of 404 patients with MDD and a follow-up sample size of 342 patients with MDD were meta-analyzed to compare depression scores before and after the intervention and at follow-up. The results showed that the depression scores at follow-up were significantly lower than those at baseline (SMD = 2.25, 95% CI (1.66, 2.84), *I*^*2*^ = 89%, *p* < 0.001; Fig. 4). Egger’s test revealed publication bias (Egger’s test, *t* = 4.27, *p* = 0.003). Meta-regression analyses were performed to assess the effects of age, sex and baseline depression severity on treatment outcomes. The results showed that age and sex had no significant effect on treatment outcomes (*p* = 0.365 for age, 0.295 for sex), while baseline depression severity had a significant positive effect on outcomes; i.e., the more severe the depression at baseline was, the greater the improvement (*p* = 0.049, *z* = 1.97).

#### Comparison of depression severity after the experiment and at follow-up

**Fig. 5:**
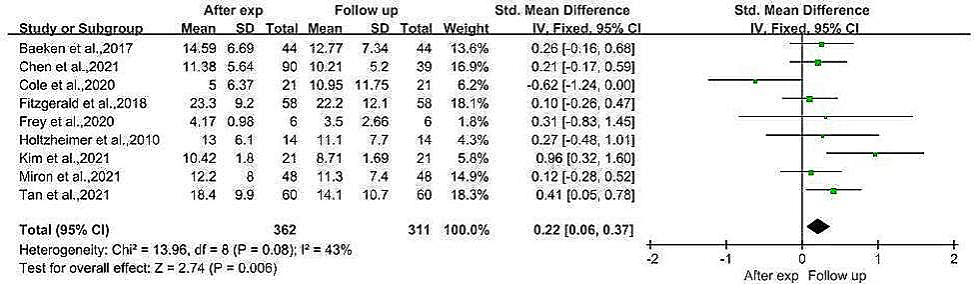
Meta analysis of depression severity after the experiment and at the follow-up

**Fig. 6:**
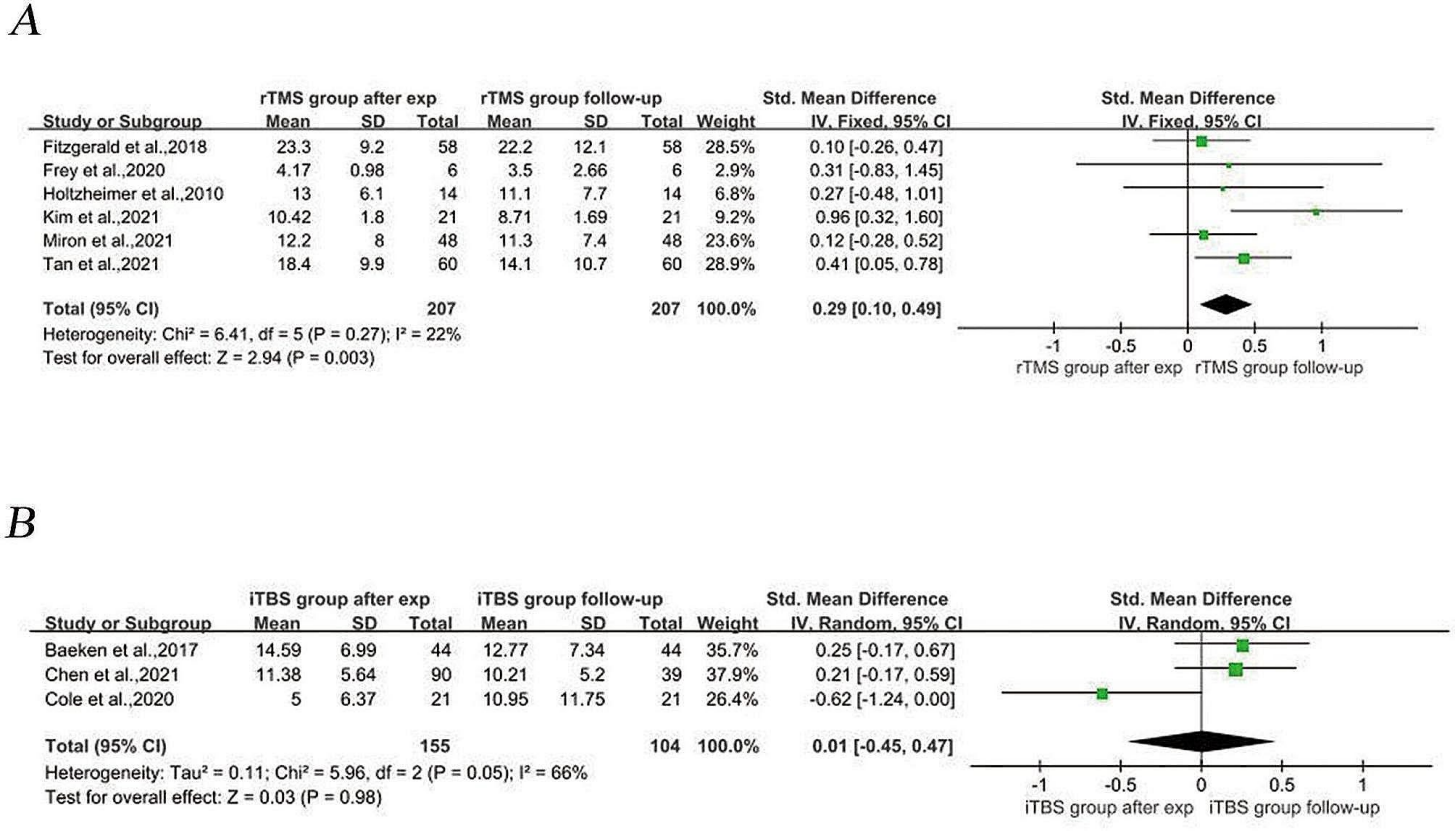
Subgroup meta-analysis comparing the severity of depression at post-experimental and follow-up in different aTMS models. 6 A: Subgroup meta-analysis of the rTMS model group. 6B: Subgroup meta-analysis of the iTBS model groupAbbreviations: rTMS: Repetitive transcranial magnetic stimulation, iTBS: intermittent theta burst stimulation, aTMS: accelerated transcranial magnetic stimulation

A meta-analysis of three RCTs and six before-and-after controlled trials with a sample size of 362 patients with MDD at posttest and 311 patients with MDD at follow-up showed that the depression scores at follow-up were lower than those directly after the intervention (SMD = 0.22, 95% CI (0.06, 0.37), *I*^*2*^ = 43%, *p* = 0.006; Fig. 5). We divided the participants into rTMS and iTBS groups according to the TMS treatment modality used. Subgroup meta-analysis comparing depression scale scores directly after the intervention and at follow-up under different TMS patterns was performed. Subgroup meta-analysis in the present study revealed that patients treated with arTMS had lower depression scale scores at follow-up than directly after the intervention (SMD = 0.29, 95% CI (0.10, 0.49), *I*^*2*^ = 22%, *p* = 0.003; Fig. 6A). In contrast, for patients receiving aiTBS, the depression scale scores did not significantly differ between follow-up and directly after the intervention (SMD = 0.01, 95% CI (−0.45, 0.47), *I*^*2*^ = 66%, *p* = 0.980; Fig. 6B). Egger’s test revealed no evidence of publication bias (*p* = 0.802). Age, sex, and the severity of depression at baseline were evaluated using meta-regression analysis to determine their effects on treatment outcomes. Age and sex had no significant effect on treatment outcome (*p* = 0.713 for age, 0.694 for sex), while baseline depression severity had a significant positive effect on outcome; i.e., the more severe the depression was at baseline, the greater the improvement (*p* = 0.03. *z* = 2.97).

#### Comparison of the efficacy of aTMS and standard TMS in improving depressive symptoms

**Fig. 7:**
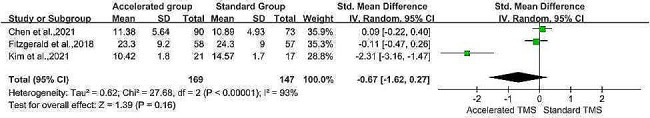
Meta-analysis of the efficacy of aTMS compared with standard TMS in improving depressive symptomsAbbreviations: aTMS: accelerated transcranial magnetic stimulation

Three RCTs were included in the meta-analysis; the experimental group (sample size: 169 patients) received aTMS, while the control group (sample size: 147 patients) received standard TMS. The L-DLPFC served as the stimulation target for both groups. Since the control group in the Baeken et al. study was sham stimulated, this study was excluded. The study’s findings showed that depression levels in the intervention and control groups were not significantly different before or after the intervention (SMD = -0.67, 95% CI (-1.62, 0.27), *I*^*2*^ = 93%, *p* = 0.160; Fig. 7). Because only three studies were included in this meta-analysis and because there was a high degree of heterogeneity in the scales used to quantify depressive symptoms, care should be taken when interpreting the results.

### Analysis of the sensitivity of the primary meta-analysis

We assessed the stability of the pooled results using the “trim-and-fill method,” which showed that the *p* value was still less than 0.05 when the meta-analysis was rerun after the inclusion of data from the sham studies, indicating that the results were robust (see Figures S1-S2 in the Supplementary Material).

Leave-one-out sensitivity analyses were also conducted for the main meta-analysis. We found that the effects of the aTMS intervention and control intervention on depressive symptoms in patients with MDD were generally reliable, with negligible effects on the meta-analysis results after excluding each study. (see Supplementary Material Table S1-S8).

## Discussion

This work is the first to perform a systematic review and meta-analysis on the therapeutic efficacy and long-term effects of arTMS and aiTBS in patients with MDD. aTMS appears to be a successful treatment for MDD according to a growing body of research [13–15]. ATMS may have rapid antidepressant effects, and its treatment outcomes are generally comparable to those of standard TMS, with no statistically significant differences. However, when contrasting aTMS with standard TMS, we included only three RCTs. Current research still lacks a large-scale study comparing aTMS to standard TMS. Does the fact that once-daily TMS requires 4–6 weeks of treatment for a response indicate that standard TMS requires a cumulative number of treatments to achieve a therapeutic effect? It is widely acknowledged that the long-term effects of four to six weeks of TMS can last up to six months after treatment [34]. By the same logic, there are likely long-term effects of accelerated TMS. This finding is consistent with the results of our meta-analysis. Additionally, we discovered that different aTMS modalities seem to have different long-term impacts. While aiTBS only sustains posttreatment effects, arTMS may continue to ameliorate depressive symptoms after treatment conclusion. The limited number of studies and the short and inconsistent follow-up durations necessitate additional experimental validation of this outcome.

### Immediate effects of aTMS on patients with MDD

For patients with MDD, aTMS is a useful treatment for reducing depression symptoms. A comparison of brain images between MDD patients and healthy participants revealed decreased activity in the DLPFC, hippocampus (HPC), and orbital frontal cortex (OFC), as well as impaired connectivity between the DLPFC and the OFC in MDD patients [35]. In clinical practice, left coil placement is frequently used, and stimulation regimens intended to treat depression typically comprise 10 Hz stimulation of the L-DLPFC [7]. This induces changes in the functional connectivity required between the L-DLPFC and the subgenual anterior cingulate cortex (sgACC) [36], and positron emission tomography (PET) revealed a significant decrease in sgACC metabolic activity among treatment responders. This finding suggests that the response to TMS may be predicted by sgACC metabolic activity at baseline [37]. Several studies have used neuroimaging techniques to investigate the effects of aTMS on depression symptoms. The accelerated HF-rTMS stimulation treatment responder group showed stronger resting-state functional connectivity between the sgACC and the left superior medial prefrontal cortex after only 4 days [36]. The gray matter volume (GMV) in the dentate gyrus of the left hippocampus was significantly increased by aTMS [23]. Following electroconvulsive treatment (ECT), comparable volume alterations were noted in the hippocampus region [38]. A correlation between GMV and depression symptoms was discovered [39]. A promising remission rate of up to 90.5% was observed with Stanford accelerated intelligent neuromodulation therapy (SAINT) for depression [11]. Furthermore, following SAINT, there was an increase in interhemispheric connectivity among the frontal gyrus, insula, and amygdala, suggesting that the therapy functions by promoting interhemispheric communication in the brain [24]. According to our meta-analysis and several brain imaging studies, patients who receive accelerated TMS can experience improvements in depression symptoms in as little as one to two weeks, which shortens the duration of treatment. However, when providing TMS, it can be difficult to prevent a placebo effect. rTMS treatment for depression has been found to significantly increase the placebo effect [40]. Attenuated suicidal ideation was linked to a considerable reduction in frontopolar prefrontal perfusion following the administration of placebo aiTBS [41]. Providing more than one treatment session per day has the potential to improve the placebo effect and lead to greater medical attention. As a core component of the default mode network (DMN), the ventromedial prefrontal cortex (vmPFC) might influence an individual’s assessment of the treatment setting and their expectation of treatment efficacy. It has a particularly important role in the placebo effect [42]. Interestingly, the stimulation area (left DLPFC) was also shown to be involved in the process of the placebo effect, whereas in most of the included studies, the stimulation sites was the L-DLPFC [43]. In addition, the placebo effect is also present in a variety of treatments that work together with real-action ingredients, contributing to the improvement of depressive symptoms.

### Long-term effects of aTMS in patients with MDD

Arici et al. reported that after rTMS treatment of patients with MDD for a total of 20 sessions over 4 weeks, 90% of those who responded remained responsive during a 6-month follow-up period [44]. According to meta-analysis of the durability of rTMS, high-frequency rTMS had a slight antidepressant effect at short-term follow-up [45]. Does aTMS’s ability to shorten treatment cycles with several daily sessions have any long-term effects? Our meta-analysis’s findings imply that this is the case. Depression scores during follow-up were lower than those directly after the intervention. This finding shows that aTMS may have a long-term effect. The results of the Duprat et al. study also indicated an increase in clinical response to 38% during the two-week follow-up after aTMS treatment [46].

Our results from a subgroup analysis comparing the efficacy of accelerated rTMS and aiTBS showed that both had good antidepressant effects. However, it seems that the delay in the effects of rTMS and iTBS might not be the same when comparing depression scale scores postintervention and follow-up. Williams et al.‘s findings imply that while aiTBS quickly reduces symptoms, depression symptoms resurface after follow-up [16]. However, in studies comparing the efficacy and follow-up of once-a-day rTMS with iTBS, no significant difference was found [47]. These differences could be attributed to the heterogeneity of the placebo effect, intervals between sessions, duration of follow-up, and Depressive Symptom Assessment Scale score. The interval between sessions may play a critical role in accelerating the cumulative effects of TMS treatment. Synapses may require multiple iTBS sessions and/or longer intervals between sessions (45–60 min) to experience the necessary plasticity mechanisms [48, 49]. Studies on rat hippocampal slices have shown that an additional long-term potentiation (LTP) effect requires a delay of 1 h between iTBS sessions [50]. Several studies have shown that aTMS intervals longer than 30 min can have a cumulative effect and induce significant cortical plasticity [51]. In our review study, session intervals ranged from 15 to 50 min, and follow-up periods ranged from 3 weeks to 3 months. The scales used to assess depressive symptoms also varied, which accounts for some of the heterogeneity in our study. Our sensitivity analyses results showed that the effects of aTMS and control interventions on depressive symptoms in patients with MDD were generally reliable. Several of the results were obtained from fewer included studies, and we need to be cautious when interpreting these results.

### Meta-regression analysis of this study

The sex ratio was found to have a positive impact on treatment efficacy according to the meta-regression study. When there is a higher number of males or a smaller number of females or when the male/female ratio is higher, treatment is more effective, indicating that aTMS may not be as successful in women as it is in men. Based on fMRI findings, males had greater neural stress responses in the right frontoparietal network (FPN) and dlPFC. However, in the networks of the amygdala, hippocampus, nucleus ambiguus (NAc), and amygdala-NAc-anterior cingulate (acc), females had more inactivation [52]. This might be connected to the fact that all of the stimulation targets in the study were in the DLPFC. Interestingly, patients who had worse depression symptoms at the beginning of treatment had better outcomes. However, current neuroscience theories have found it challenging to explain the neuronal mechanisms driving this phenomenon. This conclusion could be explained by the fact that baseline depression scale scores increase with the degree of symptom alleviation. Since several daily sessions give patients a significant amount of time for therapy and medical attention, it is possible that a stronger placebo effect would occur. In fact, the majority of the included trials did not exclude the placebo effect. Additionally, subjective patient characteristics and symptom improvement may be related.

### Limitations

Nonetheless, our study has several shortcomings. There was a lack of large RCTs in the included studies, and the sample size was small. We included only three RCTs in our analysis to determine whether aTMS or standard TMS is more effective at treating depression. Because aTMS is still a novel therapeutic modality, the available studies have varied protocols and stimulation parameters. The number of daily sessions ranged from 2 to 10 over 3–15 days, the stimulation intensities ranged from 80 to 120% of the resting motor threshold (RMT), and the total number of pulses ranged from 10,800 to 90,000. Currently, the best protocol for aTMS is unknown. Examining the impact of different models, total pulse, session intervals, and other stimulation parameters on neurophysiology and clinical outcomes is crucial. Second, the quality of the articles included in the meta-analysis was low, and the studies were mainly non-RCT studies and single-blinded RCTs. During the experiments, it was difficult to exclude the placebo effect even if the participants were blinded because of the specificity of TMS treatment. Third, regarding localization choices, all included studies focused on the DLPFC, but only four of them used precise imaging methods for localization. Accurate localization of specific targets within the DLPFC is crucial for enhancing the therapeutic efficacy of rTMS for MDD patients [53]. An incorrect stimulation target location can result in low response rates to rTMS for MDD treatment [54]. Fourth, the follow-up periods were short, ranging from 3 weeks to 3 months. In a follow-up study of standard TMS for depression, a good antidepressant effect was still observed at 6 months [44, 55]. ATMS appears to have similar long-term effects. However, long-term follow-up studies are still lacking. Fifth, in most of the original studies, the effect of medications on depressive symptoms was not excluded.

## Conclusion

In summary, aTMS improved depressive symptoms in patients with MDD more rapidly than did standard TMS. Although there seems to be no significant difference in efficacy between aTMS and standard TMS, large-scale comparative trials are still needed to validate these findings. The initial conclusion was that accelerated TMS has a long-term effect, and this effect may be related to the mode of aTMS. Specifically, the arTMS modality has sustained efficacy, whereas the aiTBS modality only maintained the effect or resulted in mild symptom worsening after treatment. However, this conclusion requires confirmation through RCTs with longer follow-up periods.

### Electronic supplementary material

Below is the link to the electronic supplementary material.

Supplementary Material 1

## Data Availability

All data generated or analysed during this study are included in this article and its supplementary information file.
