# Supplementary material for: Short-term and long-term efficacy of accelerated transcranial magnetic stimulation for depression: a systematic review and meta-analysis
Source: BMC Psychiatry. 2024 Feb 7;24:109. doi: 10.1186/s12888-024-05545-1 (PMC10851556; doi:10.1186/s12888-024-05545-1)
Supplement: Supplementary file 1 — Supplementary Material 1 [file 12888_2024_5545_MOESM1_ESM.docx]

**Short-term and Long-term efficacy of accelerated transcranial magnetism stimulation for deprsssion a systematic review and meta-analysis**

1.Search formulas：

2.Fig S1-S2：Cutting and patching method

3.Table S1-S8. “Leave-one-out” sensitivity analysis for meta-analysis

4.Fig S3. Article Bias Risk Bar Graph

5.Abbreviations

1. **Search formulas：**

Pubmed

(“Depression”[Title/Abstract] OR “Depressive Disorder”[Title/Abstract] OR “treatment-resistant depression”[Title/Abstract] OR “Major Depressive Disorder”[Title/Abstract] OR “MDD”[Title/Abstract] AND “accelerated repetitive transcranial magnetic stimulation”[Title/Abstract] OR “accelerated transcranial magnetic stimulation”[Title/Abstract] OR “accelerated rTMS”[Title/Abstract] OR “accelerated TMS”[Title/Abstract] OR “accelerated iTBS”[Title/Abstract] OR “accelerated intermittent theta burst stimulation”[Title/Abstract] OR “accelerated cTBS”[Title/Abstract] OR “accelerated continuous theta burst stimulation”[Title/Abstract] OR “Stanford Neuromodulation Therapy”[Title/Abstract])

Web Of Science

((AB=(Depression) OR AB=(Depressive Disorder) OR AB=(treatment-resistant depression) OR AB=(Major Depressive Disorder) OR AB=(MDD)) AND AB=(accelerated repetitive transcranial magnetic stimulation) OR AB=(accelerated transcranial magnetic stimulation) OR AB=(accelerated rTMS) OR AB=(accelerated TMS) OR AB=(accelerated iTBS) OR AB=(accelerated intermittent theta burst stimulation) OR AB=(accelerated cTBS) OR AB=(accelerated continuous theta burst stimulation) OR AB=(Stanford Neuromodulation Therapy))

Embase

(‘Depression’:ab,ti OR ‘Depressive Disorder’:ab,ti OR ‘Major Depressive Disorder’:ab,ti OR ‘MDD’:ab,ti) AND (‘accelerated repetitive transcranial magnetic stimulation’:ab,ti OR ‘accelerated transcranial magnetic stimulation’:ab,ti OR ‘accelerated rTMS’:ab,ti OR ‘accelerated TMS’:ab,ti OR ‘accelerated intermittent theta burst stimulation’:ab,ti OR ‘accelerated iTBS’:ab,ti OR ‘accelerated continuous theta burst stimulation’:ab,ti OR ‘accelerated cTBS’:ab,ti OR ‘Stanford Neuromodulation Therapy’:ab,ti)

1. **Fig S1**: Cutting and patching method


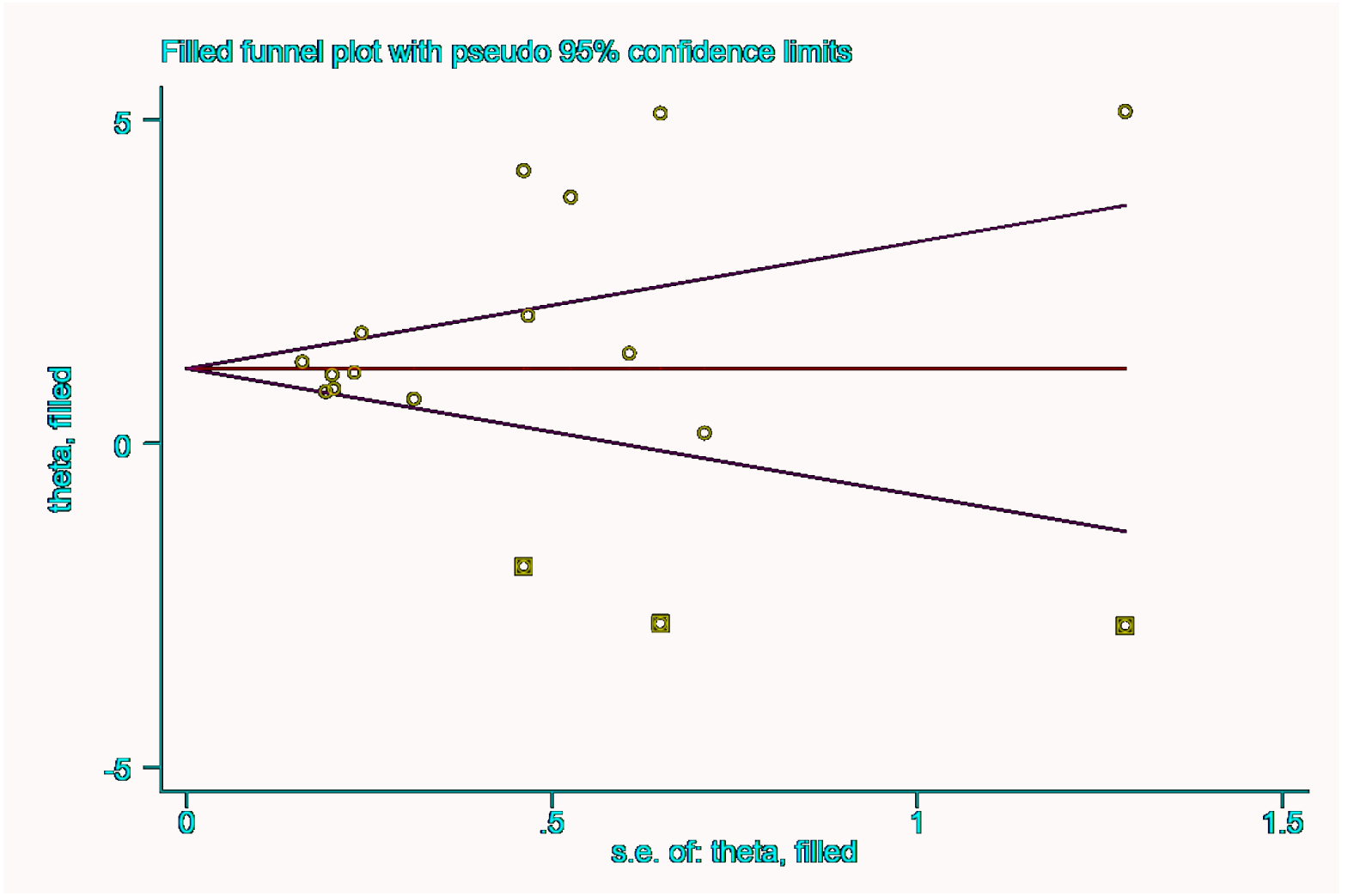


Reliability analysis of the Egger's test revealed publication bias (Egger's test, t = 2.76, p = 0.017).

**Fig S2：**Cutting and patching method


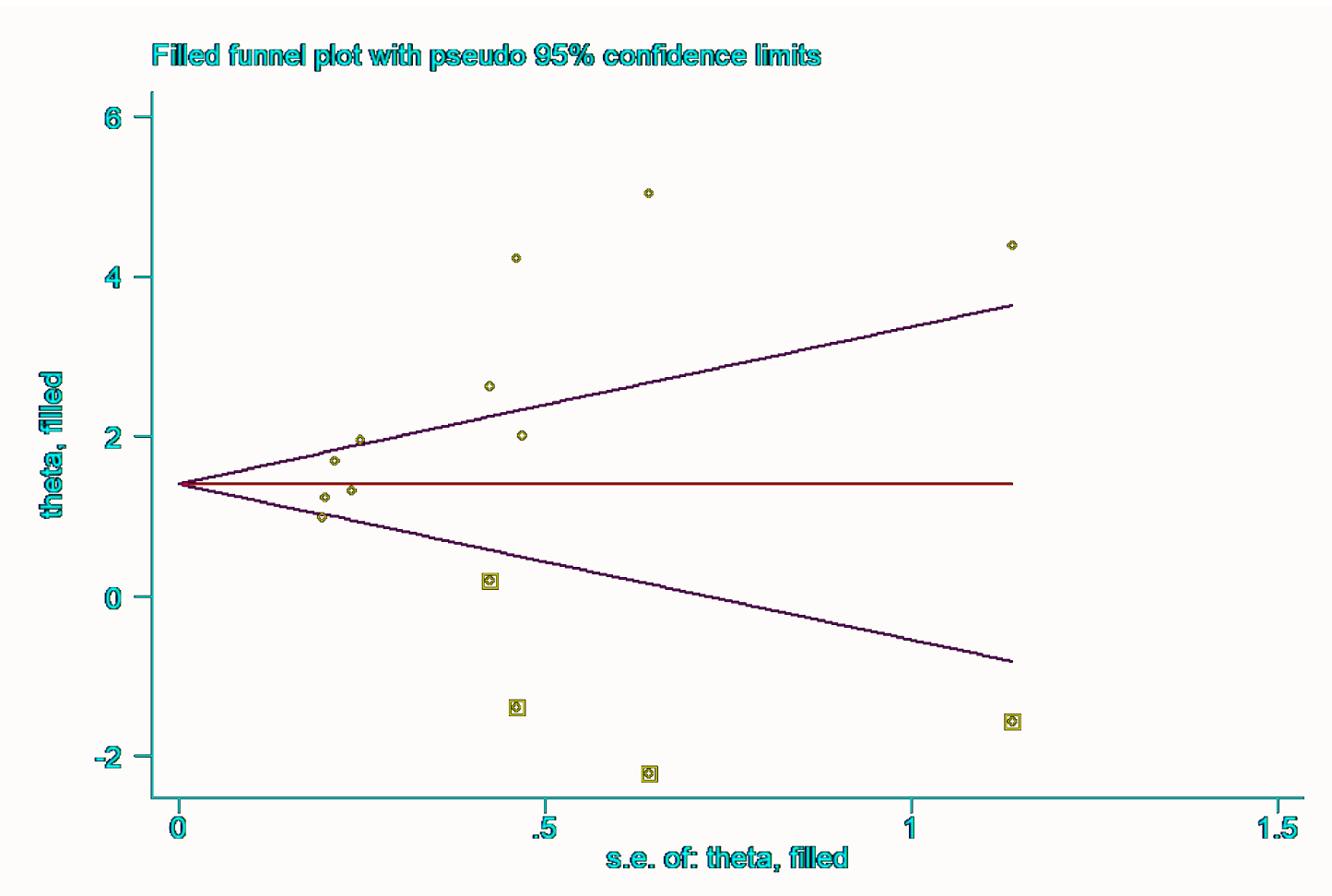


Reliability analysis of the Egger's test revealed publication bias (Egger's test, t = 4.27, *p* = 0.003).

1. **Table S1**："Leave-one-out" sensitivity analysis for inclusion in the study's pre- and post-experimental meta-analysis

| Excluded Study,Year | Country | SMD (95% CI) | *P* value | N case | N control | Test of heterogeneity (*P*/ *I^2^*) |
| --- | --- | --- | --- | --- | --- | --- |
| Baeken et al., 2017 | Belgium | 1.88 (1.34，.43) | <0.00001 | 447 | 436 | <0.00001/90% |
| Zhang et al., 2022 | China | 1.57 (1.14，2.01) | <0.00001 | 460 | 449 | <0.00001/86% |
| Baeken et al., 2020 | Belgium | 1.91 (1.83，2.43) | <0.00001 | 469 | 458 | <0.00001/90% |
| Tor et al., 2016 | Australia | 1.84 (1.32，2.35) | <0.00001 | 484 | 473 | <0.00001/90% |
| Tan et al., 2021 | Singapore | 1.91 (1.37，2.46) | <0.00001 | 431 | 420 | <0.00001/90% |
| Miron et al., 2021 | Canada | 1.83 (1.29，2.36) | <0.00001 | 443 | 432 | <0.00001/90% |
| Kim et al., 2021 | Korea | 1.65 (1.17，2.21) | <0.00001 | 470 | 459 | <0.00001/88% |
| Jodoin et al., 2019 | USA | 1.91 (1.36，2.45) | <0.00001 | 437 | 426 | <0.00001/90% |
| Cantù et al., 2021 | Italy | 1.90 (1.39，2.40) | <0.00001 | 487 | 476 | <0.00001/90% |
| Cole et al., 2020 | USA | 1.58 (1.13，2.03) | <0.00001 | 470 | 459 | <0.00001/87% |
| Fitzgerald et al., 2018 | Australia | 1.89 (1.34，2.45) | <0.00001 | 433 | 422 | <0.00001/90% |
| Holtzheimer et al., 2010 | USA | 1.80 (1.28，2.31) | <0.00001 | 477 | 466 | <0.00001/90% |
| Frey et al., 2020 | USA | 1.72 (1,23，2.21) | <0.00001 | 485 | 474 | <0.00001/90% |
| Chen et al.,(2021) | Australia | 1.88 (1.31，2.46) | <0.00001 | 390 | 390 | <0.00001/90% |

SMD, standardized mean difference; CI, confidence interval; N cases, number of cases; N controls, number of controls.

| Excluded Study,Year | Country | SMD (95% CI) | *P* value | N case | N control | Test of heterogeneity (*P*/ *I^2^*) |
| --- | --- | --- | --- | --- | --- | --- |
| Baeken et al., 2017 | Belgium | 2.40 (1.72，3.07) | <0.00001 | 360 | 298 | <0.00001/90% |
| Fitzgerald et al., 2018 | Australia | 2.43 (1.79，3.07) | <0.00001 | 346 | 284 | <0.00001/89% |
| Cole et al., 2020 | USA | 2.21 (1.59，2.84） | <0.00001 | 383 | 321 | <0.00001/90% |
| Holtzheimer et al., 2010 | USA | 2.29 (1.65，2.92) | <0.00001 | 390 | 328 | <0.00001/91% |
| Frey et al., 2020 | USA | 2.16 (1.57，2.75) | <0.00001 | 398 | 336 | <0.00001/90% |
| Zhang et al., 2022 | China | 1.98 (1.46，2.49) | <0.00001 | 373 | 311 | <0.00001/85% |
| Tan et al., 2021 | Singapore | 2.41 (1.73，3.09) | <0.00001 | 344 | 282 | <0.00001/90% |
| Kim et al., 2021 | Korea | 1.97 (1.45，2.50) | <0.00001 | 383 | 321 | <0.00001/86% |
| Chen et al.,(2021) | Australia | 2.35 (1.66，3.04) | <0.00001 | 303 | 303 | <0.00001/91% |
| Miron et al., 2021 | Canada | 2.31 (1.64，2.99） | <0.00001 | 356 | 294 | <0.00001/90% |

1. **Table S2**："Leave-one-out" sensitivity analysis for meta-analysis comparing pre-experimental and follow-up

SMD, standardized mean difference; CI, confidence interval; N cases, number of cases; N controls, number of controls.

3**.Table S3**："Leave-one-out" sensitivity analysis for meta-analyses comparing post-experimental and follow-up

| Excluded Study,Year | Country | SMD (95% CI) | *P* value | N case | N control | Test of heterogeneity (*P*/ *I^2^*) |
| --- | --- | --- | --- | --- | --- | --- |
| Baeken et al., 2017 | Belgium | 0.21 (0.04，0.38) | 0.050 | 318 | 267 | 0.01/50% |
| Fitzgerald et al., 2018 | Australia | 0.24 (0.07，0.41) | 0.060 | 304 | 253 | 0.006/48% |
| Cole et al., 2020 | USA | 0.27 (0.11，0.43) | 0.480 | 341 | 290 | 0.0009/0% |
| Holtzheimer et al., 2010 | USA | 0.21 (0.06，0.37) | 0.050 | 348 | 297 | 0.008/50% |
| Frey et al., 2020 | USA | 0.21 (0.06，0.37) | 0.050 | 356 | 305 | 0.007/50% |
| Tan et al., 2021 | Singapore | 0.17 (0.00，0.34） | 0.080 | 302 | 251 | 0.05/44% |
| Kim et al., 2021 | Korea | 0.17 (0.01，0.33) | 0.290 | 341 | 290 | 0.04/17% |
| Chen et al.,(2021) | Australia | 0.22 (0.05，0.39) | 0.050 | 272 | 272 | 0.01/50% |
| Miron et al., 2021 | Canada | 0.23 (0.07，0.40) | 0.060 | 314 | 263 | 0.006/49% |

SMD, standardized mean difference; CI, confidence interval; N cases, number of cases; N controls, number of controls.

3.**Table S4**："Leave-one-out" sensitivity analysis for meta-analysis comparing aTMS and standard TMS

| Excluded Study,Year | Country | SMD (95% CI) | *P* value | N case | N control | Test of heterogeneity (*P*/ *I^2^*) |
| --- | --- | --- | --- | --- | --- | --- |
| Kim et al., 2021 | Korea | 0.01 (-0.23，0.24) | 0.410 | 148 | 130 | 0.95/0% |
| Chen et al.,(2021) | Australia | -1.18 (-3.34，0.98) | <0.00001 | 79 | 74 | 0.29/95% |
| Fitzgerald et al., 2018 | Australia | -1.08 (-3.43，1.28) | <0.00001 | 111 | 90 | 0.37/96% |

SMD, standardized mean difference; CI, confidence interval; N cases, number of cases; N controls, number of controls; ATMS, accelerated transcranial magnetic stimulation; TMS, transcranial magnetic stimulation.

| Excluded Study,Year | Country | SMD (95% CI) | *P* value | N case | N control | Test of heterogeneity (*P*/ *I^2^*) |
| --- | --- | --- | --- | --- | --- | --- |
| Baeken et al., 2017 | Belgium | 2.22 (0.81，3.64) | <0.00001 | 179 | 168 | 0.002/95% |
| Baeken et al., 2020 | Belgium | 2.28 (1.05，3.51) | <0.00001 | 201 | 190 | 0.0003/94% |
| Chen et al.,(2021) | Australia | 2.18 (0.64，3.73) | <0.00001 | 122 | 122 | 0.006/95% |
| Cantù et al., 2021 | Italy | 2.29 (1.19，3.40) | <0.00001 | 219 | 208 | <0.00001/95% |
| Cole et al., 2020 | USA | 1.47 (0.59，2.36) | <0.00001 | 202 | 191 | 0.001/91% |
| Zhang et al., 2022 | China | 1.52 (0.65，2.38) | <0.00001 | 192 | 181 | 0.0006/90% |

3.**Table S5**：ITBS group: "leave-one-out" sensitivity analysis comparing pre-experimental and follow-up subgroup meta-analysis

ITBS, intermittent theta burst stimulation; SMD, standardized mean difference; CI, confidence interval; N cases, number of cases; N controls, number of controls.

3.**Table S6**：RTMS group: "leave-one-out" sensitivity analysis comparing pre-experimental and follow-up subgroup meta-analysis

| Excluded Study,Year | Country | SMD (95% CI) | *P* value | N case | N control | Test of heterogeneity (*P*/ *I^2^*) |
| --- | --- | --- | --- | --- | --- | --- |
| Fitzgerald et al., 2018 | Australia | 1.80 (1.10，2.49) | <0.00001 | 210 | 210 | <0.00001/87% |
| Frey et al., 2020 | USA | 1.50 (0.97，2.02) | <0.00001 | 262 | 262 | <0.00001/84% |
| Holtzheimer et al., 2010 | USA | 1.60 (1.00，2.19） | <0.00001 | 254 | 254 | <0.00001/86% |
| Jodoin et al., 2019 | USA | 1.82 (1.16，2.48) | <0.00001 | 214 | 214 | <0.00001/85% |
| Tor et al., 2016 | Australia | 1.68 (1.08，2.27) | <0.00001 | 261 | 261 | <0.00001/87% |
| Tan et al., 2021 | Singapore | 1.83 (1.17，2.48) | <0.00001 | 208 | 208 | <0.00001/85% |
| Miron et al., 2021 | Canada | 1.64 (1.01，2.27) | <0.00001 | 220 | 220 | <0.00001/85% |
| Kim et al., 2021 | Korea | 1.29 (0.87，1.70) | 0.0020 | 247 | 247 | <0.00001/72% |

RTMS, Repetitive transcranial magnetic stimulation; SMD, standardized mean difference; CI, confidence interval; N cases, number of cases; N controls, number of controls.

3.**Table S7**: ITBS group: "leave-one-out" sensitivity analysis comparing post-experimental and follow-up subgroup meta-analysis

| Excluded Study,Year | Country | SMD (95% CI) | *P* value | N case | N control | Test of heterogeneity (*P*/ *I^2^*) |
| --- | --- | --- | --- | --- | --- | --- |
| Baeken et al., 2017 | Belgium | -0.17 (-0.97，0.64) | 0.69 | 111 | 60 | 0.03/80% |
| Chen et al.,(2021) | Australia | -0.15 (-1.00，0.70) | 0.73 | 65 | 65 | 0.02/81% |
| Cole et al., 2020 | USA | 0.23 (-0.05，0.51) | 0.11 | 134 | 83 | 0.98/0% |

ITBS; intermittent theta burst stimulation; SMD, standardized mean difference; CI, confidence interval; N cases, number of cases; N controls, number of controls.

3.**Table S8**: RTMS group: "leave-one-out" sensitivity analysis comparing post-experimental and follow-up subgroup meta-analysis

| Excluded Study,Year | Country | SMD (95% CI) | *P* value | N case | N control | Test of heterogeneity (*P*/ *I^2^*) |
| --- | --- | --- | --- | --- | --- | --- |
| Fitzgerald et al., 2018 | Australia | 0.37 (0.14，0.60) | 0.002 | 149 | 149 | 0.29/19% |
| Frey et al., 2020 | USA | 0.29 (0.09，0.49) | 0.004 | 201 | 201 | 0.17/38% |
| Holtzheimer et al., 2010 | USA | 0.29 (0.09，0.50） | 0.004 | 193 | 193 | 0.17/38% |
| Tan et al., 2021 | Singapore | 0.24 (0.01，0.47) | 0.04 | 147 | 147 | 0.22/31% |
| Miron et al., 2021 | Canada | 0.35 (0.12，0.57) | 0.002 | 159 | 159 | 0.25/26% |
| Kim et al., 2021 | Korea | 0.22 (0.02，0.43) | 0.03 | 186 | 186 | 0.77/0% |

RTMS, Repetitive transcranial magnetic stimulation; SMD, standardized mean difference; CI, confidence interval; N cases, number of cases; N controls, number of controls.

1. **Fig S3**. Article Bias Risk Bar Graph


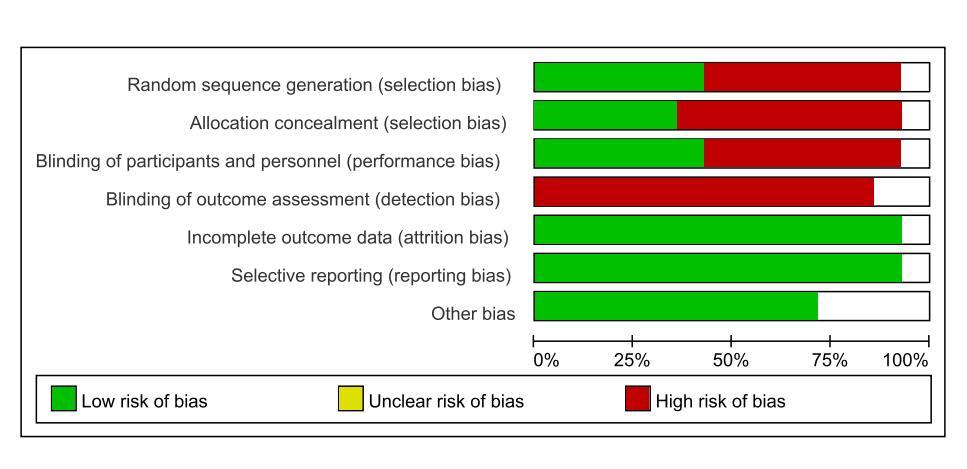


1. **Abbreviations**：

Abbreivations: TMS: Transcranial magnetic stimulation; aTMS: accelerated transcranial magnetic stimulation; SrTMS: Standard repetitive transcranial magnetic stimulation; MT: motor threshold; iTBS: intermittent theta burst stimulation; cTBS: continuous theta burst stimulation; L-DLPFC: left dorsolateral prefrontal cortex; R-DLPFC: Right dorsolateral prefrontal cortex; EPDS: Edinburgh Postnatal Depression Scale; HAMD: Hamilton Rating Scale for Depression; MADRS: Montgomery Åsberg Depression Rating Scale; MDD: Major depressive disorder; TRD: treatment-resistant depression; BD: Bipolar Disorder; HAM-A: Hamilton Rating Scale for Anxiety; BDI-II: Beck Depression Inventory II; HRSD-17: 17-item Hamilton Rating Scale for Depression; KQIDS-SR: Korean Quick Inventory of Depressive Symptomatology Self-reported; KQIDS-C: Korean Quick Inventory of Depressive Symptomatology Clinician administered; SSI: scale of suicidal ideation; BDI: Beck depression inventory; BHS: Beck Hopelessness Scale; QIDS-SR16: Quick Inventory of Depressive Symptomatology-Self-Rated Version; QIDS-C16: Quick Inventory of Depressive Symptomatology-Clinician Rated Version; SSI: Scale for Suicidal Ideation; RCTs: randomized controlled trials; HPC: hippocampus; OFC: orbital frontal cortex; sgACC: subgenual anterior cingulate cortex; GMV: Gray matter volume; ECT: electroconvulsive therapy; SAINT: Stanford Accelerated Intelligent Neuromodulation Therapy; vmPFC: ventromedial prefrontal cortex; DMN: default mode network; RMT: resting motor threshold; PPD: postpartum depression; PSD: post-stroke depression.
